# Supplementary material for: Mechanism of Action of Antitumor Au(I) N-Heterocyclic Carbene Complexes: A Computational Insight on the Targeting of TrxR Selenocysteine
Source: Int J Mol Sci. 2024 Feb 23;25(5):2625. doi: 10.3390/ijms25052625 (PMC10932012; doi:10.3390/ijms25052625)
Supplement: Supplementary file 1 [file ijms-25-02625-s001.zip › ijms-2896407-supplementary.pdf]

International Journal of *Molecular Sciences*

MDPI

Supporting Information for:

Mechanism of Action of Antitumor Au(I) N-Heterocyclic Carbene Complexes: A Computational Insight on the Targeting of TrxR Selenocysteine

Iogann Tolbatov [1,*], Paolo Umari [1] and Alessandro Marrone [2]

[1] Department of Physics and Astronomy, University of Padova, Via F. Marzolo 8, 35131 Padova, Italy; paolo.umari@unipd.it
[2] Dipartimento di Farmacia, Università "G d'Annunzio" di Chieti-Pescara, Via dei Vestini 31, 66100 Chieti, Italy; amarrone@unich.it
* Correspondence: tolbatov.i@gmail.com

Cartesian coordinates for calculated transition state structures for the reaction of complex 1 with ethylselenolate via different mechanisms:

<u>**No assistance**</u>:

Au	0.16600	0.29100	-0.01200
C	3.63600	-2.01500	-1.00600
C	4.15500	-1.24700	-0.01500
N	3.14200	-0.40800	0.40500
C	1.99600	-0.63400	-0.28600
N	2.31600	-1.63200	-1.14800
H	5.15000	-1.22500	0.41900
C	-3.21400	-2.55000	1.19900
C	-3.83900	-1.96700	0.14500
N	-2.90100	-1.15600	-0.46600
C	-1.70400	-1.21300	0.16400
N	-1.91300	-2.08200	1.18200
H	-4.86000	-2.05800	-0.21400
H	4.08900	-2.79100	-1.61600
H	-3.58100	-3.25000	1.94500
C	3.26000	0.56700	1.48100
H	4.31600	0.87300	1.53400
H	2.66300	1.44200	1.18500
C	2.77600	0.02400	2.81600
C	1.39900	-2.16000	-2.14800
H	1.70300	-3.19400	-2.36600
H	0.40200	-2.19000	-1.68400
C	1.37700	-1.31300	-3.41100
C	-3.16100	-0.30900	-1.62800
C	-2.36800	-0.73900	-2.85000
H	-4.24200	-0.35300	-1.82300
H	-2.90400	0.72600	-1.35400
C	-0.91900	-2.35800	2.20700

C	-0.97500	-1.35200	3.34700
H	-1.07900	-3.38500	2.57100
H	0.06400	-2.32600	1.71500
C	0.64900	3.83300	-1.29700
C	-0.02100	3.81900	0.06800
Se	-1.25800	2.31600	0.36100
H	1.26000	2.92700	-1.43500
H	0.73600	3.80300	0.86600
H	-0.62000	4.73300	0.20900
H	-0.10100	3.85100	-2.10300
H	1.30300	4.71500	-1.41300
H	-2.62400	-0.10100	-3.70700
H	-1.29200	-0.62600	-2.65500
H	-2.57300	-1.78700	-3.11700
H	-0.19300	-1.57000	4.08900
H	-0.82200	-0.33300	2.95900
H	-1.95000	-1.38200	3.85600
H	2.89300	0.78700	3.59900
H	1.70900	-0.23400	2.75000
H	3.34100	-0.87200	3.11400
H	0.65700	-1.72400	-4.13300
H	1.07700	-0.28300	-3.16900
H	2.36800	-1.28300	-3.88900

Phosphate buffer assistance:

Au	0.59500	-0.47700	0.03000
C	-2.58900	-3.33700	0.32500
C	-2.23800	-3.25000	1.63100
N	-1.18600	-2.35600	1.69300
C	-0.87800	-1.87500	0.46300
N	-1.74900	-2.49200	-0.37300
H	-2.63900	-3.74000	2.51300
C	1.27800	3.78300	1.25400
C	1.02400	4.01200	-0.06000
N	0.53400	2.82900	-0.57700
C	0.48300	1.86000	0.36800
N	0.93400	2.46800	1.49200
H	1.14500	4.91300	-0.65400
H	-3.35800	-3.92600	-0.16600
H	1.66200	4.44000	2.02900
C	-0.60600	-1.88100	2.94200
H	-0.46900	-2.74600	3.60700
H	0.39300	-1.49100	2.70300
C	-1.47200	-0.81300	3.59300
C	-1.82300	-2.29600	-1.82000
H	-2.80200	-2.68500	-2.13200
H	-1.84300	-1.21100	-2.00100
C	-0.70600	-2.99800	-2.57400
C	0.12400	2.64000	-1.96600
C	-0.86300	3.69700	-2.43200
H	1.02600	2.62500	-2.59800
H	-0.36700	1.65900	-2.01000
C	1.09500	1.80400	2.77800
C	2.54400	1.46000	3.08300
H	0.66900	2.45500	3.55700
H	0.48000	0.89700	2.73600
C	3.12300	-2.91600	-1.71800

C	3.90300	-1.84800	-0.96800
Se	3.00300	-0.09700	-0.79300
H	2.19300	-3.17100	-1.18700
H	4.15500	-2.20700	0.04100
H	4.85500	-1.64600	-1.48200
P	-3.37100	1.03800	-0.60800
O	-3.90000	2.59800	-0.76900
O	-2.45700	0.80000	-1.78500
O	-4.55800	0.16500	-0.31800
O	-2.47800	1.13400	0.76800
H	-1.52300	1.15800	0.56200
H	-4.68400	2.72600	-0.22400
H	2.84800	-2.57500	-2.72800
H	3.71800	-3.83900	-1.81900
H	-1.16600	3.47500	-3.46600
H	-1.76600	3.67700	-1.80500
H	-0.43400	4.71000	-2.42200
H	2.60700	0.91900	4.03800
H	2.96000	0.82700	2.28500
H	3.16000	2.36800	3.16500
H	-0.96800	-0.40100	4.47900
H	-1.67500	-0.00000	2.87900
H	-2.43800	-1.23200	3.91300
H	-0.84700	-2.85900	-3.65600
H	0.27300	-2.58000	-2.30200
H	-0.69600	-4.07800	-2.36300

Reaction with the CysSec peptide:

Au	1.55300	-0.52600	0.39400
C	5.78800	-0.75600	0.01300
C	5.68400	0.58500	0.18800
N	4.33900	0.85800	0.34000
C	3.60000	-0.27500	0.28200
N	4.50600	-1.26300	0.08100
H	6.44500	1.36000	0.21500
C	0.26100	2.45400	-3.10000
C	0.20000	3.29200	-2.03600
N	0.36600	2.50600	-0.91000
C	0.52100	1.19600	-1.23100
N	0.45800	1.18600	-2.58300
H	0.04500	4.36600	-1.99800
C	3.78600	2.17900	0.60500
H	4.50400	2.91800	0.22100
H	2.86900	2.27100	0.00900
C	3.50500	2.40400	2.08300
C	4.16100	-2.66900	-0.09800
H	5.03400	-3.26200	0.21100
H	3.33800	-2.89000	0.59700
C	3.75400	-2.99100	-1.52700
C	0.21000	2.96600	0.46500
C	0.72300	4.37600	0.69500
H	-0.85900	2.91000	0.71600
H	0.73900	2.23900	1.09700
C	0.49400	-0.02800	-3.38500
C	-0.83600	-0.30800	-4.07100
H	1.31000	0.05600	-4.12100
H	0.75800	-0.83900	-2.69200

Se	-0.59400	-1.61600	1.09600
H	0.67900	4.60900	1.76900
H	1.76400	4.49700	0.35800
H	0.10200	5.12200	0.17800
H	-0.80100	-1.28400	-4.57600
H	-1.66300	-0.31300	-3.34600
H	-1.06300	0.45400	-4.83200
H	3.08200	3.40600	2.24000
H	2.78400	1.65900	2.45200
H	4.42500	2.31900	2.68000
H	3.51200	-4.06000	-1.61800
H	2.86100	-2.41300	-1.80600
H	4.56100	-2.75600	-2.23700
N	-2.83500	1.30300	1.33500
C	-3.96400	1.27300	0.42600
C	-3.68400	0.17900	-0.61400
N	-3.55500	-1.05600	-0.07300
O	-3.55400	0.41000	-1.80400
C	-2.92000	1.89900	2.55700
C	-4.32400	2.62300	-0.19800
S	-2.99800	3.50200	-1.07400
H	-4.85800	0.96900	1.00300
H	-4.63600	3.29200	0.61400
H	-5.17800	2.48300	-0.87500
H	-2.77900	2.54300	-1.99600
H	-2.01400	0.73100	1.12800
C	-2.84900	-2.10600	-0.78100
C	-3.39300	-3.47500	-0.33100
O	-4.48100	-3.85200	-0.72200
N	-2.59800	-4.19500	0.49800
H	-3.53400	-1.11100	0.94000
C	-1.33600	-1.87200	-0.71900
H	-3.16100	-2.03000	-1.83300
H	-1.12100	-0.94700	-1.27100
H	-0.80800	-2.69700	-1.22000
C	-3.00000	-5.47700	1.02100
H	-1.74400	-3.76600	0.85300
H	-2.23800	-6.24500	0.81600
H	-3.16800	-5.43400	2.10900
H	-3.93900	-5.76300	0.53100
O	-3.92100	2.48300	2.93000
C	-1.66200	1.80900	3.39700
H	-1.94900	1.63900	4.44300
H	-0.98100	1.01500	3.05900
H	-1.14000	2.77700	3.35000
H	6.65800	-1.38700	-0.14900
H	0.18100	2.65600	-4.16500
